# AMACR and ZFPL1 serum biomarkers enhance precision in predicting postoperative prostate cancer outcomes

**DOI:** 10.3389/fonc.2026.1625125

**Published:** 2026-02-17

**Authors:** Lin Yang, Renguang Lv, Zhao Liu, Gang Chen, Gangli Gu

**Affiliations:** 1 Department of Urology, Qilu Hospital, Shandong University, Jinan, China; 2 Department of Urology, Jinan Seventh People’s Hospital, Jinan, China

**Keywords:** postoperative prognosis, prediction model, prostate cancer, risk assessment, zinc fingerprotein-like 1, α-Methylacyl-CoA racemase

## Abstract

**Background:**

Prostate cancer (PCa) remains a major clinical challenge, with postoperative recurrence risk varying substantially among patients. Emerging evidence suggests that serum biomarkers, including α-methylacyl-CoA racemase (AMACR) and zinc finger protein-like 1 (ZFPL1), may provide additional prognostic information beyond conventional clinicopathological factors.

**Materials and methods:**

This single-center retrospective study included 115 patients with PCa who underwent radical prostatectomy. Serum AMACR and ZFPL1 levels were analyzed in combination with clinicopathological variables. Univariate and multivariate logistic regression analyses were performed to identify independent predictors of recurrence. A risk nomogram was constructed, and model performance was evaluated using receiver operating characteristic (ROC) curve analysis and Kaplan–Meier survival analysis.

**Results:**

Poor postoperative outcomes were significantly associated with advanced age, lymph node metastasis, higher TNM stage, poor tumor differentiation, and higher Gleason score. Serum AMACR and ZFPL1 levels were significantly elevated in patients who experienced recurrence. Multivariate analysis identified both biomarkers as independent predictors of recurrence. The resulting nomogram demonstrated strong discriminative performance and stable predictive accuracy across multiple postoperative time points.

**Conclusion:**

Elevated serum AMACR and ZFPL1 levels independently predict recurrence following radical prostatectomy in patients with PCa. The proposed nomogram integrates molecular and clinicopathological factors to provide accurate postoperative risk stratification, supporting individualized follow-up and management, with robust performance observed up to 3 years after surgery.

## Graphical Abstract

**Figure:**
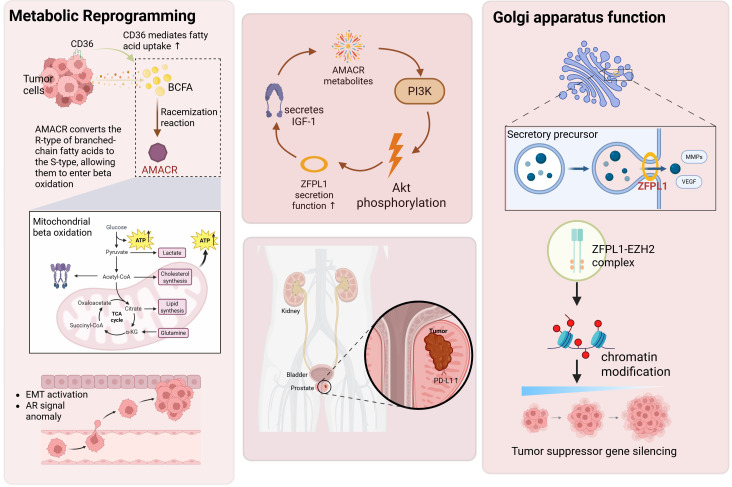
The molecular mechanism by which AMACR and ZFPL1 regulate postoperative recurrence in PCa.

## Introduction

Prostate cancer (PCa) is the most common malignant tumor of the male genitourinary system, with over 1.4 million new cases diagnosed globally each year. Postoperative recurrence and metastasis remain major clinical challenges (1, 2). Radical prostatectomy (RP) is the primary treatment for localized PCa and significantly improves patient survival; however, approximately 20%-40% of patients still develop biochemical recurrence or distant metastasis after surgery (3–5). Currently, commonly used prognostic assessment tools, such as the CAPRA score and TNM staging system, are largely based on histopathological characteristics (e.g., Gleason score, surgical margin status) and the dynamic changes in prostate-specific antigen (PSA) levels (6). However, these indicators often show limited sensitivity and substantial interindividual heterogeneity in predicting postoperative recurrence risk (7). Therefore, the identification of novel biomarkers and the development of more accurate prognostic models are of critical clinical importance.

In recent years, non-invasive biomarkers based on liquid biopsies have emerged as a research hotspot in PCa. α-Methylacyl-CoA racemase (AMACR), a PCa-specific metabolic enzyme, is highly expressed in tumor tissues and positively correlated with tumor aggressiveness (8). Recent studies indicate that AMACR can be detected in various bodily fluids such as urine and semen (9), and that elevated serum AMACR levels are significantly associated with early biochemical recurrence after surgery (10), suggesting its potential as a prognostic biomarker. Zinc finger protein-like 1 (ZFPL1) is a Golgi-localized zinc-finger domain protein that plays an important role in maintaining Golgi structural integrity and secretory function. Previous studies have reported aberrant overexpression of ZFPL1 in various malignancies, where it may promote tumor cell proliferation, invasion, and apoptosis dysregulation through pathways such as PI3K–Akt (11, 12). Previous research (13) suggests that combining AMACR with other molecular markers, such as CD15 and PSMA, significantly improves postoperative risk stratification. Meanwhile, ZFPL1, a protein associated with neuroendocrine phenotype, may provide novel insights into predicting castration-resistant progression. However, no studies have systematically evaluated the prognostic significance of combined serum AMACR and ZFPL1 detection for long-term outcomes following RP in PCa patients. Their incremental contribution within multivariate predictive models also remains unclear.

In recent years, the widespread adoption of precision medicine in urologic oncology has transformed postoperative management of PCa from a traditional “watchful waiting” approach to a strategy focused on risk stratification and individualized intervention. However, prognostic tools commonly used in clinical practice, such as the CAPRA score and Gleason grading, show limited sensitivity for the early detection of residual disease after surgery and often fail to support dynamic monitoring or personalized follow-up in patients at high risk of recurrence (14, 15). Meanwhile, global PCa research is increasingly moving toward an integrated framework combining multi-omics analyses and liquid biopsy technologies. Several prospective cohorts in Europe and North America are actively investigating the prognostic value of noninvasive circulating biomarkers, including serum-based markers, exosomes, and circulating tumor DNA (ctDNA), for predicting postoperative recurrence (16–18). Therefore, the development of predictive models based on serum molecular biomarkers not only aligns with international research trends but also offers substantial potential to improve the precision and individualization of postoperative clinical decision-making.

Given the challenges in accurately predicting postoperative recurrence and the limited sensitivity of existing prognostic tools, we hypothesized that serum AMACR and ZFPL1 could serve as independent predictors of PCa recurrence following surgery, based on their distinct biological characteristics. Accordingly, we performed a retrospective analysis of 115 patients who underwent RP to evaluate the associations between these biomarkers and recurrence-free survival (RFS), and to construct a risk nomogram integrating serum biomarkers with key clinicopathological variables.

## Materials and methods

### Study population

This single-center retrospective cohort study was conducted in accordance with the Reporting Recommendations for Tumor Marker Prognostic Studies (REMARK) guidelines. A total of 115 patients with PCa who underwent RP at our institution between January 2018 and January 2021 were retrospectively enrolled. All diagnoses were pathologically confirmed postoperatively in accordance with the *2022 CSCO Prostate Cancer Guidelines* (19), and written informed consent was obtained from all participants (Version V3.0). Based on postoperative follow-up data over a 3-year period, patients were classified into two groups: a favorable outcome group (n = 85), defined as no evidence of recurrence, and an unfavorable outcome group (n = 30), defined as the occurrence of biochemical recurrence, radiographic metastasis, or all-cause mortality within 3 years after surgery.

**Figure 1:**
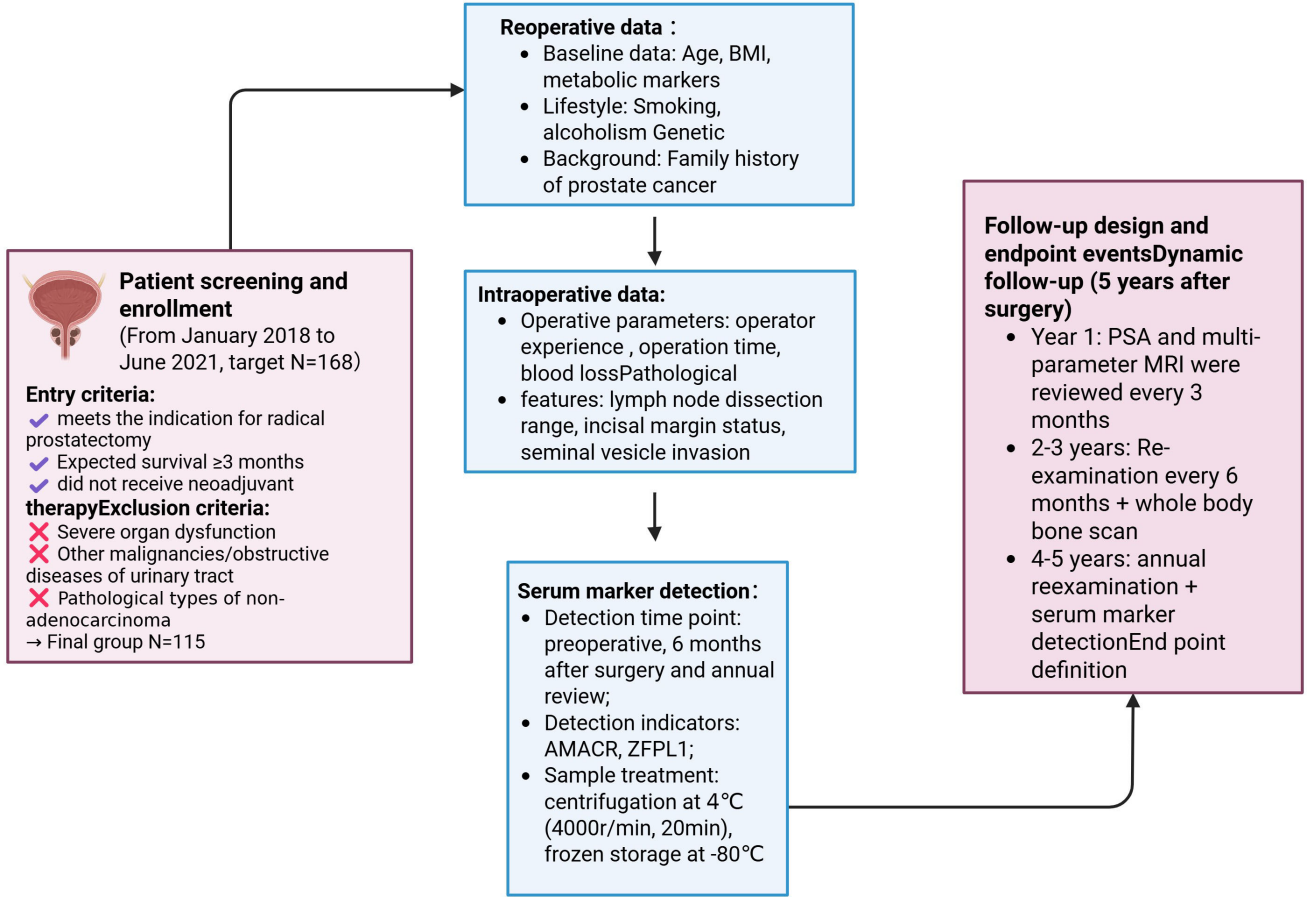
Study flowchart.

The inclusion criteria were as follows: (1) eligibility for RP (clinical stage ≤ T3aN0M0); (2) expected survival time ≥ 3 months; (3) no neoadjuvant endocrine therapy, radiotherapy, or chemotherapy before surgery; and (4) availability of complete clinical and follow-up data. Exclusion criteria included: (1) severe major organ dysfunction (Child-Pugh class ≥ C for liver function, eGFR < 30 mL/min/1.73 m^2^, or NYHA class ≥ III for cardiac function); (2) presence of other concurrent malignancies (second primary cancer or metastatic tumors); (3) significant obstructive urinary tract diseases (e.g., neurogenic bladder, urinary tract stones, or strictures); (4) psychiatric disorders that could compromise follow-up compliance; and (5) non-adenocarcinoma histology on postoperative pathology (e.g., squamous cell carcinoma, neuroendocrine carcinoma). A flowchart illustrating patient selection is presented in **Figure 1**.

The sample size was determined by consecutive enrollment, including all patients who underwent RP during the study period and met the inclusion/exclusion criteria (n = 115). As this study was exploratory in nature, no formal *a priori* sample size estimation or statistical power calculation was performed. Because the analysis was retrospective and based on existing clinical data, the study protocol was not prospectively registered in a public clinical trial registry. Nevertheless, the study was approved by the institutional ethics committee and conducted in strict accordance with the *Declaration of Helsinki*. All patient information was anonymized prior to analysis.

### Clinical data collection and extended variables

To enhance the clinical applicability of the predictive model, this study expanded preoperative and intraoperative variables beyond routine baseline data, including age, body mass index (BMI), maximum tumor diameter, TNM stage, and Gleason score. Preoperative variables included lifestyle factors—heavy smoking (defined as a smoking index ≥ 20 pack-years) and alcohol abuse (defined as weekly ethanol consumption > 50 g), and family history of PCa or other malignant tumors in first-degree relatives. Metabolism-related indicators, including fasting blood glucose, total cholesterol, and triglyceride levels, were also collected. Intraoperative variables comprised surgical team experience (defined as a primary surgeon having performed more than 100 RP procedures), operative duration (from skin incision to wound closure), intraoperative blood loss (recorded to the nearest 50 mL), extent of lymph node dissection (extended dissection defined as en bloc removal of internal iliac, external iliac, and obturator lymph nodes), surgical margin status (R0 indicating microscopically negative margins and R1 indicating microscopically positive margins), and seminal vesicle invasion confirmed by postoperative pathology. By integrating multidimensional clinical and procedural data, this study aimed to comprehensively evaluate potential interaction effects between serum biomarkers and clinical interventions.

### Serum biomarker detection and standardization procedures

Fasting peripheral venous blood samples (3 mL) were collected from each patient preoperatively and at 6 months postoperatively. Serum was separated by centrifugation at 4 °C and 4,000 rpm for 20 minutes using a Beckman Coulter Avanti J-26S centrifuge, aliquoted into Eppendorf tubes, and stored at −80 °C until analysis. Serum concentrations of AMACR and ZFPL1 were measured using double-antibody sandwich enzyme-linked immunosorbent assay (ELISA) kits (Beijing Wode Biotechnology Co., Ltd.; catalog numbers WD-AMACR-2020 and WD-ZFPL1-2021). Each assay batch included high-, medium-, and low-concentration quality control samples. Both intra-assay and inter-assay coefficients of variation (CV) were maintained below 5%, ensuring assay reliability and reproducibility.

### Long-term follow-up and endpoint definition

Patients were followed dynamically for up to three years after surgery. Follow-up visits were scheduled every three months during the first postoperative year and every six months during the second and third years. Assessments included serial PSA measurements, with baseline PSA established at 6 weeks postoperatively, multiparametric magnetic resonance imaging (performed at 1 year after surgery and upon abnormal PSA elevation), annual whole-body bone scans, and repeated measurements of serum AMACR and ZFPL1 levels at 6 months postoperatively and annually thereafter. The primary endpoint was 3-year RFS, defined as the interval from surgery to biochemical recurrence (PSA ≥ 0.2 ng/mL with confirmed sustained elevation) or radiographic evidence of metastasis. Secondary endpoints included overall survival (OS) and longitudinal changes in serum biomarker levels. Patients lost to follow-up were censored at the time of last contact in Kaplan–Meier analyses, and reasons for loss to follow-up, such as relocation or withdrawal of consent, were documented.

### Model development and validation

A prognostic model incorporating serum AMACR and ZFPL1 levels was developed and validated using data from 115 postoperative PCa patients with complete 3-year recurrence outcomes and biomarker profiles. The cohort was randomly divided into a training set (n = 81) and a test set (n = 34) at a 7:3 ratio using stratified sampling to preserve outcome proportions (caret v6.0-94, R v4.2.2). A multivariable logistic regression model was built in the training set, incorporating AMACR, ZFPL1, TNM stage, Gleason score, and tumor differentiation to predict recurrence within 3 years. Model performance was assessed by area under the ROC curve (AUC) using the pROC package (v1.18.0), with 95% confidence intervals estimated via the DeLong method. To evaluate concordance between predicted risk and actual recurrence, Kaplan-Meier curves stratified by predicted risk were generated in the test set. The overall workflow is illustrated in **Supplementary Figure S1**.

### Public data acquisition and differential expression analysis

RNA sequencing data processed using the STAR pipeline and reported in transcripts per million (TPM) format were obtained from the TCGA Prostate Adenocarcinoma (TCGA-PRAD) cohort via the Genomic Data Commons portal (https://portal.gdc.cancer.gov). Expression profiles were extracted for tumor and normal prostate tissues. The unpaired cohort consisted of 501 tumor and 52 normal samples, while the paired cohort comprised 52 matched tumor-normal pairs based on sample identifiers. Differential expression in unpaired samples was assessed using the Mann-Whitney U test, whereas paired comparisons were performed using the Wilcoxon signed-rank test. Expression distributions and paired differences were visualized using violin plots and connected dot plots generated with ggplot2 (v3.4.2). All statistical tests were two-sided, and significance levels were indicated using conventional asterisk notation.

### Immunohistochemistry images from the human protein atlas database

Immunohistochemical (IHC) staining images and expression annotations for AMACR and ZFPL1 in normal and tumor prostate tissues were retrieved from the HPA database (https://www.proteinatlas.org). Images were generated using antibody HPA015527 for AMACR and antibody HPA044909 for ZFPL1. Tissue staining was performed by the pathology platform at Uppsala University in accordance with standardized HPA protocols, and image quality and expression scoring were curated by the HPA expert panel.

### Statistical analysis

All statistical analyses were performed using SPSS (version 25.0) and R (version 4.3.1). Continuous variables are presented as mean ± standard deviation (SD) or median with interquartile range (IQR), depending on data distribution assessed by the Shapiro–Wilk test. Categorical variables are summarized as counts and percentages. Between-group comparisons were conducted using Student’s *t*-test or the Mann–Whitney *U* test for continuous variables and the chi-square test or Fisher’s exact test for categorical variables.

Univariate logistic regression was used to identify factors associated with unfavorable outcomes. Variables with *p <* 0.10 in univariate analysis were entered into a multivariate logistic regression model to identify independent predictors. Serum AMACR and ZFPL1 levels were analyzed as continuous variables in all regression and ROC analyses, without dichotomization or predefined cutoff values. A prognostic nomogram was then constructed based on multivariate results, incorporating AMACR, ZFPL1, TNM stage, Gleason score, tumor differentiation, and age. Model discrimination was evaluated using the area under the receiver operating characteristic curve (AUC), with 95% confidence intervals calculated *via* the DeLong method. Model calibration was assessed using calibration plots and the Hosmer-Lemeshow goodness-of-fit test. Internal validation was performed with 1,000 bootstrap resampling iterations to assess model stability and potential overfitting. Concordance index (C-index) was also calculated to quantify overall discriminative ability.

Time-dependent ROC curves at 1, 2, and 3 years were generated using the timeROC package to assess dynamic predictive accuracy. Clinical utility was evaluated using decision curve analysis (DCA). Recurrence-free survival was analyzed using the Kaplan–Meier method and compared between groups using the log-rank test. All statistical tests were two-sided, and p < 0.05 was considered statistically significant. No missing data were present for modeling variables; therefore, imputation procedures were not required.

## Results

### Univariate analysis of clinicopathological features and the impact of AMACR and ZFPL1 on postoperative prognosis in PCa

**Figure 2:**
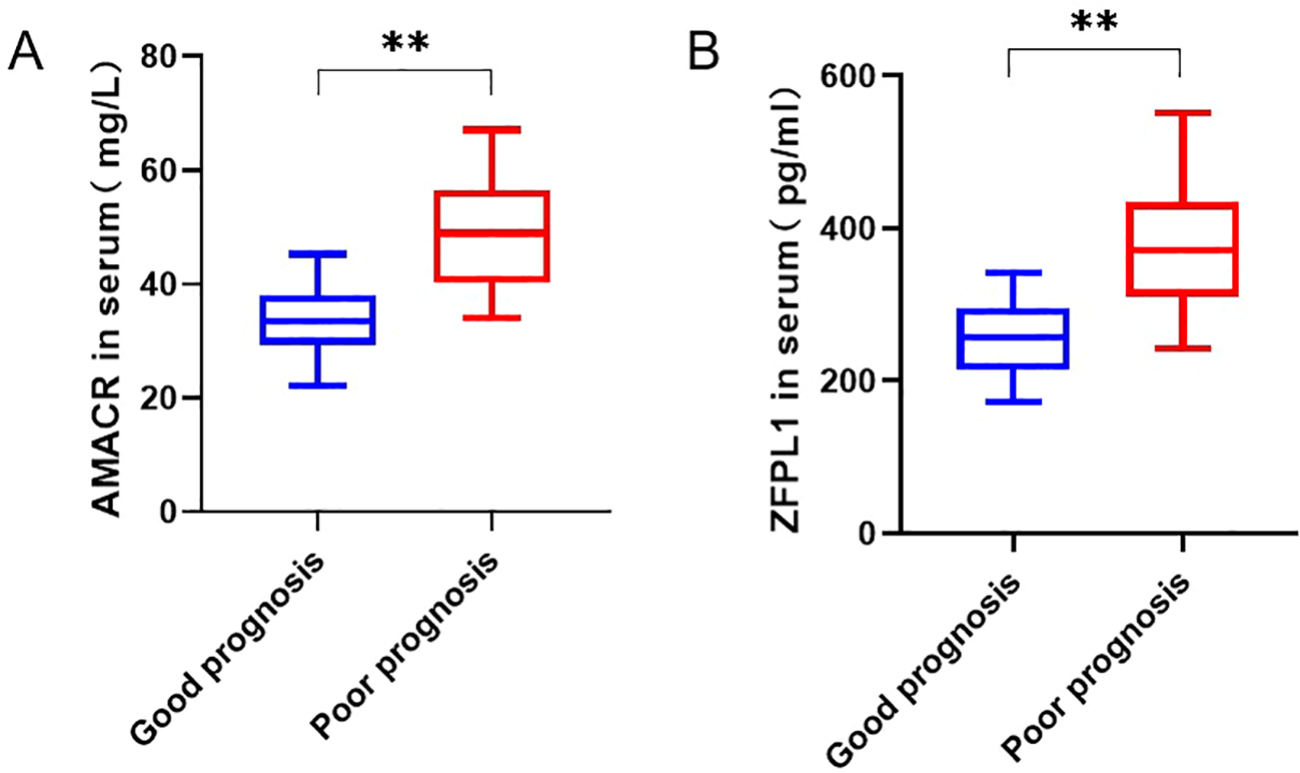
Serum levels of AMACR and ZFPL1. **(A)** Comparison of serum AMACR levels between prognosis groups. **(B)** Comparison of serum ZFPL1 levels between prognosis groups. ZFPL1 levels in the poor prognosis group (red boxplots, n = 30) are significantly higher than those in the favorable prognosis group (blue boxplots, n = 85) (**p < 0.001).

nivariate analysis demonstrated that, compared with patients without recurrence (n = 85), those who experienced recurrence (n = 30) had significantly higher proportions of age ≥ 60 years (60.0% vs. 37.6%, χ^2^ = 4.508, *p* = 0.034), lymph node metastasis (60.0% vs. 35.3%, χ^2^ = 5.566, *p* = 0.018), TNM stage III-IV (66.7% vs. 43.5%, χ^2^ = 4.749, *p* = 0.029), poor tumor differentiation (73.3% vs. 38.8%, χ^2^ = 10.585, *p* = 0.005), and Gleason score ≥ 8 (66.7% vs. 41.2%, χ^2^ = 5.774, *p* = 0.016) (**Table 1**). Serum biomarker analysis revealed significantly elevated AMACR levels in the recurrence group (48.2 mg/L, IQR: 42.5-54.1) compared with the non-recurrence group (32.3 mg/L, IQR: 26.8-38.9; Mann-Whitney U = 217, *p <* 0.001). Similarly, serum ZFPL1 levels were markedly higher in patients with recurrence (374.3 pg/mL, IQR: 305.4-443.2) than in those without recurrence (238.3 pg/mL, IQR: 189.5-287.1; Mann-Whitney U = 185, *p <* 0.001) (**Figures 2A, B**). No significant differences are observed between groups regarding BMI, tumor diameter, or other variables (all *p* > 0.05). In contrast, other baseline clinical characteristics, including BMI, maximum tumor diameter, prostate volume, pathological subtype, histories of diabetes and hypertension, smoking status, and alcohol consumption, did not differ significantly between the two groups (all *p* > 0.05) (**Table 1**).

**Table 1:** Univariate analysis of poor prognosis after prostate cancer surgery [n (%); mean ± standard deviation].

Factor	Good prognosis croup (n=85)	Poor prognosis group (n=30)	χ^2^/t	P
Age			4.508	0.034
≥60 years	32 (37.65)	18 (60.00)		
<60 years	53 (62.35)	12 (40.00)		
BMI			0.188	0.665
≥22.81kg/m^2^	35 (41.18)	11 (36.67)		
<22.81kg/m^2^	50 (58.82)	19 (63.33)		
Maximum tumor diameter			0.052	0.820
≥5cm	32 (37.65)	12 (40.00)		
<5cm	53 (62.35)	18 (60.00)		
Prostate volume			0.487	0.485
≥35ml	28 (32.94)	12 (40.00)		
<35ml	57 (67.06)	18 (60.00)		
Pathological type			0.368	0.985
Acinar Adenocarcinoma	75 (88.24)	27 (90.00)		
Intraductal Carcinoma	5 (5.88)	1 (3.33)		
Ductal Adenocarcinoma	3 (3.53)	1 (3.33)		
Other	2 (2.35)	1 (3.33)		
Lymph node metastasis			5.566	0.018
Yes	30 (35.29)	18 (60.00)		
No	55 (64.71)	12 (40.00)		
Lymph node metastasis			0.406	0.524
Yes	28 (32.94)	8 (26.67)		
No	57 (67.03)	22 (73.33)		
TNM staging			4.749	0.029
I~II	48 (56.47)	10 (33.33)		
III~IV	37 (43.53)	20 (66.67)		
Differentiation grade			10.585	0.005
Poorly Differentiated	33 (38.82)	22 (73.33)		
Moderately Differentiated	32 (37.65)	5 (16.67)		
Well Differentiated	20 (23.53)	3 (10.00)		
Gleason score			5.774	0.016
≥8	35 (41.18)	20 (66.67)		
<8	50 (58.82)	10 (33.33)		
Diabetes			0.082	0.774
Yes	15 (17.65)	6 (20.00)		
No	70 (82.35)	24 (80.00)		
Hypertension			1.779	0.182
Yes	18 (21.18)	10 (33.33)		
No	67 (78.82)	20 (66.67)		
Smoking			0.375	0.540
Yes	48 (56.47)	15 (50.00)		
No	37 (43.53)	15 (50.00)		
Alcohol Consumption			1.604	0.205
Yes	51 (60.00)	14 (46.67)		
No	34 (40.00)	16 (53.33)		
Serum AMACR (mg/L)	32.33 ± 8.75	48.26 ± 10.33	8.170	<0.001
Serum ZFPL1 (pg/ml)	238.34 ± 56.72	374.29 ± 85.66	9.792	<0.001

### Multivariate analysis of independent risk factors for poor postoperative prognosis in PCa patients

Variables with *p* < 0.05 in univariate analyses were included in the multivariable logistic regression model. Age, lymph node metastasis, TNM stage, pathological differentiation, Gleason score, and serum AMACR and ZFPL1 levels were entered into the model. The analysis identified age ≥ 60 years (OR = 1.802, 95% CI: 1.092–3.377), TNM stage III–IV (OR = 2.034, 95% CI: 1.167–4.858), poor tumor differentiation (OR = 1.984, 95% CI: 1.168–4.150), Gleason score ≥8 (OR = 1.996, 95% CI: 1.101–4.027), elevated serum AMACR (OR = 1.910, 95% CI: 1.086–3.943), and elevated serum ZFPL1 levels (OR = 1.902, 95% CI: 1.116–3.578) as independent predictors of recurrence (**Table 2**). Variable coding is shown in **Table 3**. The model demonstrated good calibration (Hosmer–Lemeshow χ² = 7.210, *p* = 0.514), indicating an adequate model fit.

**Table 2:** Logistic regression analysis of postoperative poor prognosis in prostate cancer patients.

Factor	β	S.E.	Waldχ^2^	*P*-value	OR value	95%CI
Age	0.589	0.213	7.647	<0.001	1.802	1.092~3.377
Lymph Node Metastasis	0.526	0.387	1.847	0.185	1.692	1.123~2.859
Clinical Stage	0.710	0.308	5.314	<0.001	2.034	1.167~4.858
Differentiation Grade	0.685	0.264	6.732	<0.001	1.984	1.168~4.150
Gleason Score	0.691	0.312	4.905	<0.001	1.996	1.101~4.027
AMACR	0.647	0.289	5.012	<0.001	1.910	1.086~3.943
ZFPL1	0.643	0.256	6.309	<0.001	1.902	1.116~3.578

**Table 3:** Assignment of independent variables.

Variable	Factor	Assignment
Dependent Variable	Prognosis	Good = 0, Poor = 1
Independent Variable	Age	<60 years = 0, ≥60 years = 1
Independent Variable	Lymph Node Metastasis	No = 0, Yes = 1
Independent Variable	Clinical Stage	Stage I–II = 0, Stage III–IV = 1
Independent Variable	Differentiation Grade	Well-differentiated = 0, Moderately-differentiated = 1, Poorly-differentiated = 2
Independent Variable	Gleason Score	<8 = 0, ≥8 = 1
Independent Variable	AMACR	Actual value
Independent Variable	ZFPL1	Actual value

### Construction of a risk nomogram model for poor postoperative prognosis in PCa patients

**Figure 3:**
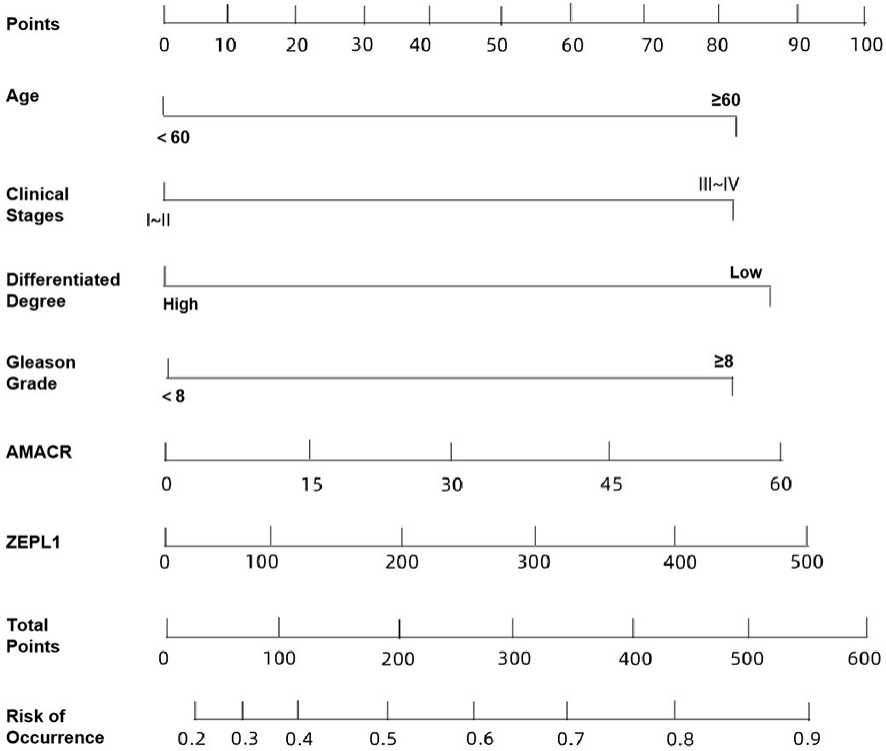
Risk nomogram model constructed for independent risk factors of poor postoperative prognosis in PCa patients.

A risk nomogram was developed based on the multivariable logistic regression model using the training cohort (n = 81) to estimate the probability of recurrence within 3 years after surgery (**Figure 3**). TNM stage III–IV contributed the highest number of points (35), followed by Gleason score ≥ 8 (30 points), poor differentiation (25 points), and age ≥ 60 years (20 points). Each standard deviation increase in serum AMACR and ZFPL1 levels contributed 15 and 10 points, respectively.

**Figure 4:**
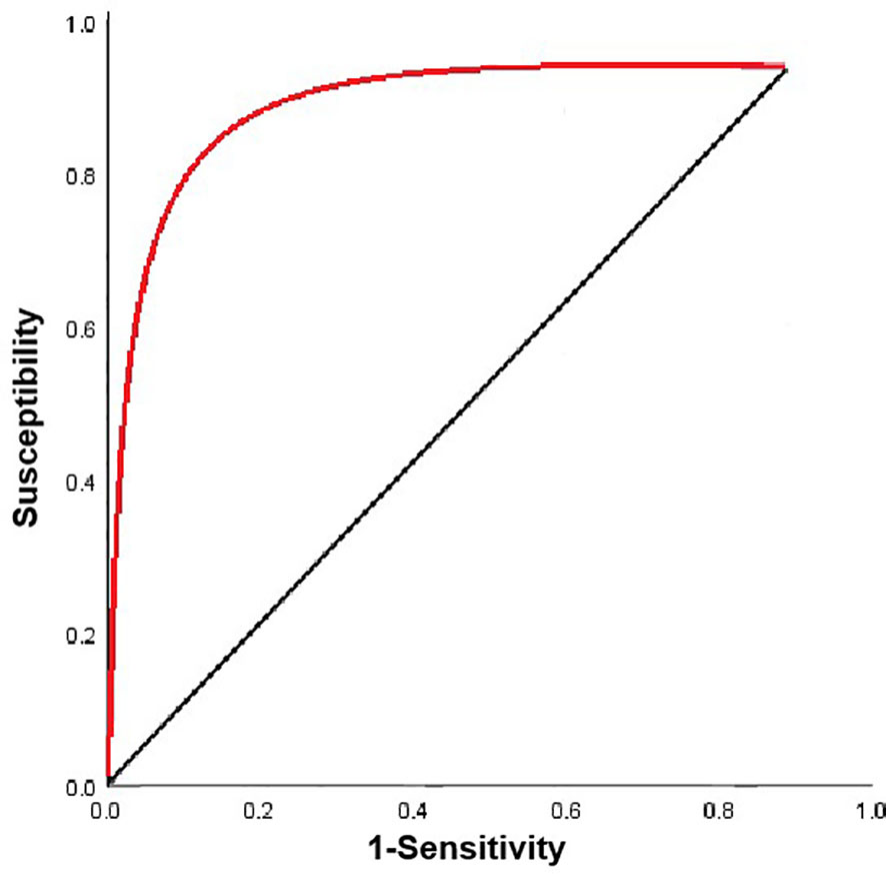
ROC curve of the risk nomogram model based on serum AMACR and ZFPL1 levels for predicting poor postoperative prognosis in PCa patients.

**Figure 5:**
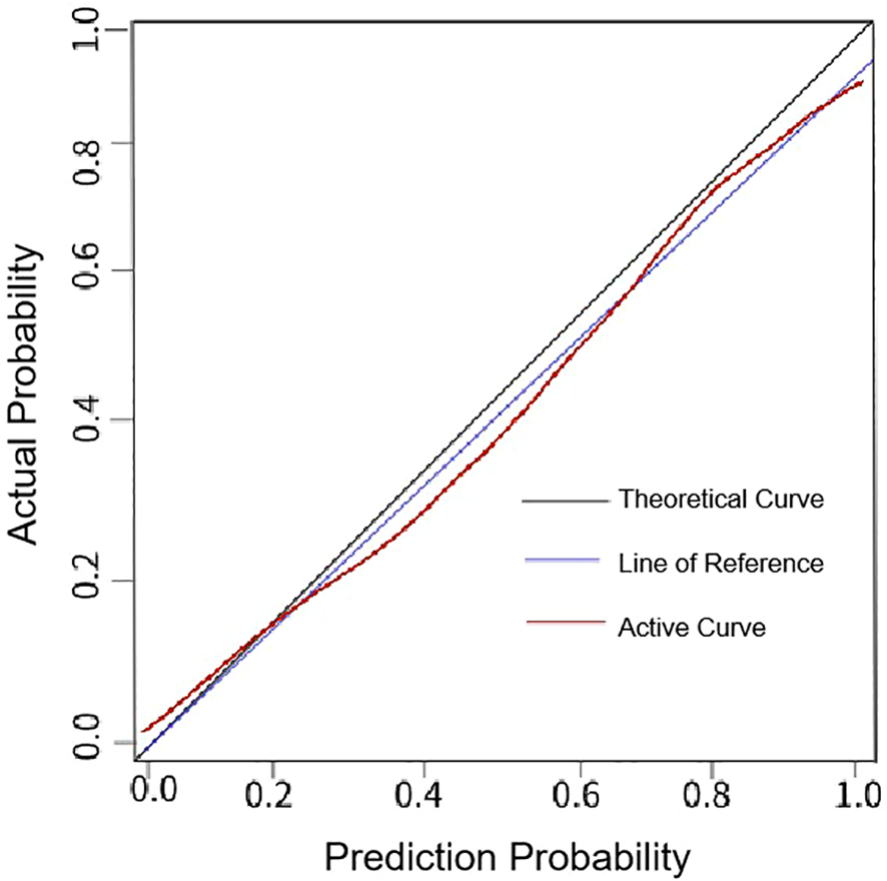
Calibration curve of the risk nomogram model based on serum AMACR and ZFPL1 levels for predicting poor postoperative prognosis in PCa patients.

Model performance evaluation demonstrated strong discrimination, with an AUC of 0.872 (95% CI: 0.788-0.952), sensitivity of 90.00% (27/30), specificity of 92.94% (79/85), and overall accuracy of 92.17% (106/115) (**Figure 4**). Calibration analysis showed good agreement between predicted and observed recurrence probabilities, with a Brier score of 0.11 and Hosmer-Lemeshow χ^2^ = 3.356 (P = 0.318), indicating satisfactory calibration (**Figure 5**).

**Figure 6:**
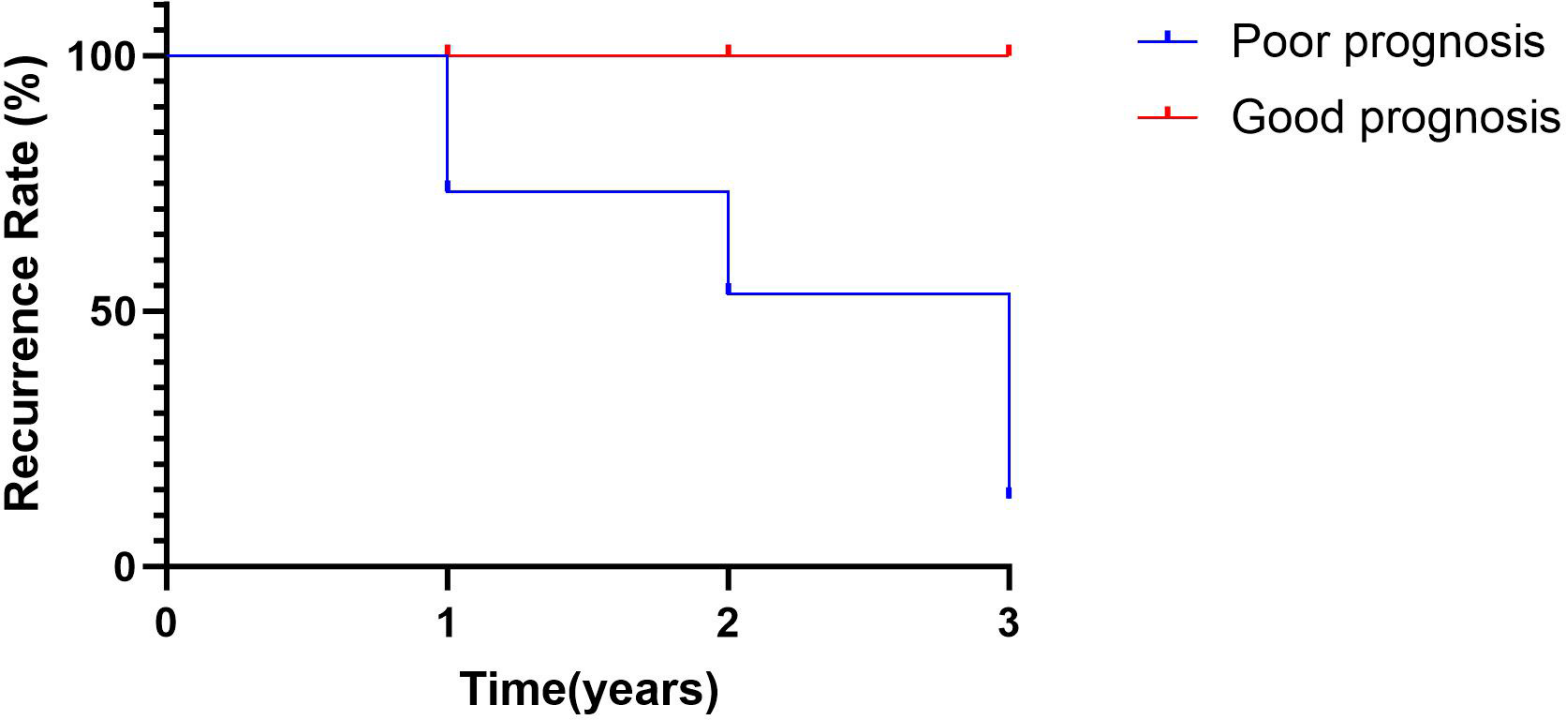
ROC curves comparing the nomogram and PSAV for predicting poor postoperative prognosis after radical prostatectomy. **p < 0.001.

For comparison, a model based solely on postoperative PSA kinetics, using PSA velocity measured between 3 and 12 months after surgery, yielded an AUC of 0.759 (95% CI: 0.649–0.870). At the optimal cutoff value of 0.19 ng/mL/year, this model achieved a sensitivity of 80.0% (24/30), specificity of 65.9% (56/85), and accuracy of 69.6% (80/115). Although the nomogram showed a numerically higher AUC than the PSA-based model (0.872 vs. 0.760), the difference did not reach statistical significance (DeLong test Z = 1.67, *p* = 0.095; 95% CI for AUC difference: −0.021 to 0.258) (**Figure 6**). These results suggest a potential advantage of the integrated nomogram, which warrants further validation in larger, independent cohorts.

### Time-dependent predictive performance of the model and validation of biomarker levels

**Figure 7:**
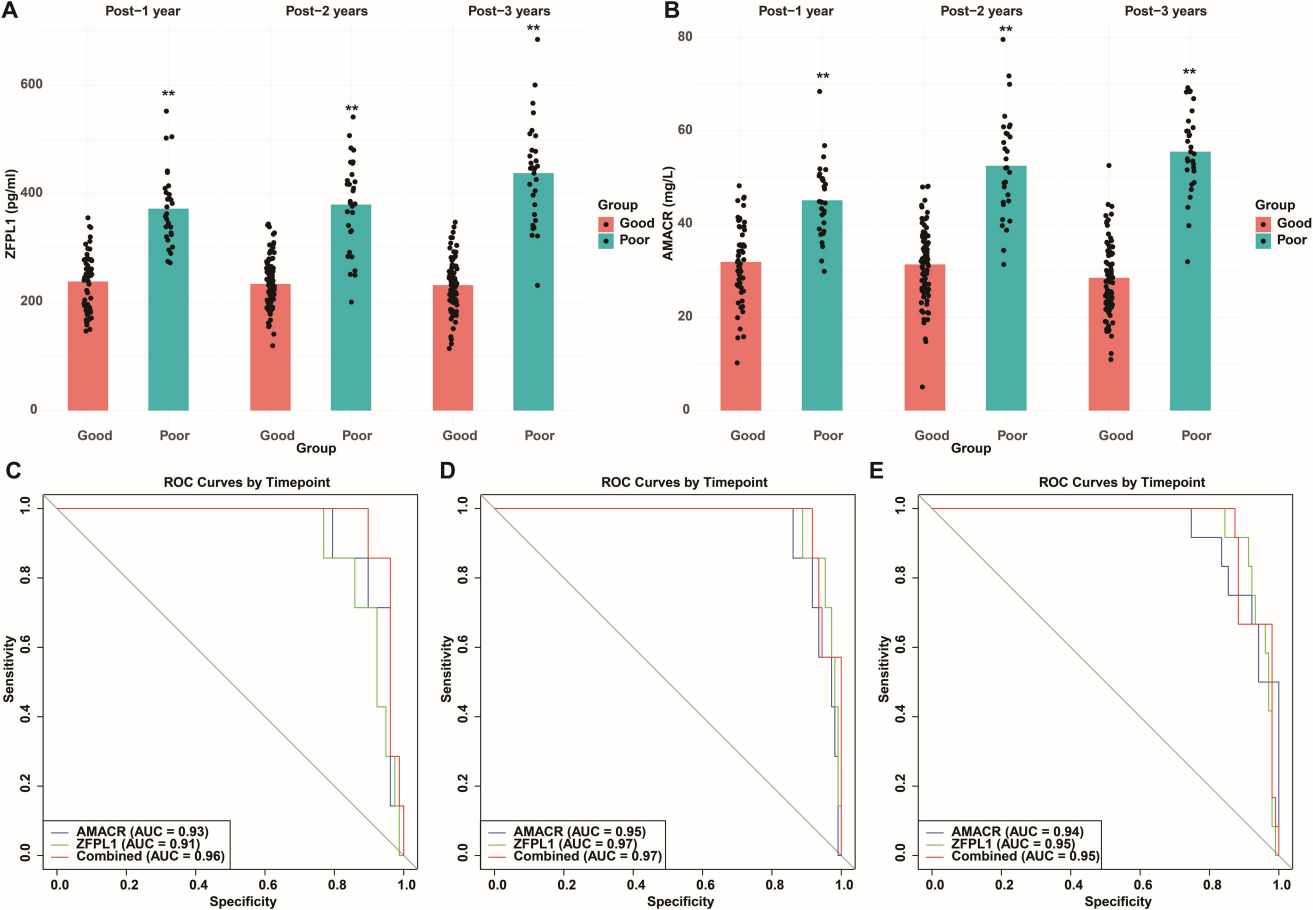
Evaluation of the time-dependent prognostic performance of the nomogram model based on serum AMACR and ZFPL1 levels. **(A)** Bar and scatter plots of ZFPL1 levels between favorable and poor prognosis groups at 1 year postoperatively. **(B)** Bar and scatter plots of AMACR levels between favorable and poor prognosis groups at 1 year postoperatively. **(C)** ROC curves for prognosis prediction at 1 year postoperatively: AUC = 0.93 (AMACR), AUC = 0.91 (ZFPL1), and AUC = 0.96 (combined model). **(D)** ROC curves for prognosis prediction at 2 years: AUC = 0.95 (AMACR), AUC = 0.97 (ZFPL1), and AUC = 0.97 (combined model). **(E)** ROC curves for prognosis prediction at 3 years: AUC = 0.94 (AMACR), AUC = 0.96 (ZFPL1), and AUC = 0.95 (combined model).

The nomogram incorporating serum AMACR and ZFPL1 levels was evaluated for time-dependent predictive performance at 1, 2, and 3 years after surgery. Serum levels of both biomarkers were consistently and significantly higher in patients who experienced recurrence than in those without recurrence, and these differences remained stable across all postoperative time points (*p* < 0.01) (**Figures 7A, B**).

Time-dependent ROC curves and corresponding AUC values at 1, 2, and 3 years after surgery were used to evaluate predictive performance. The combined model incorporating both AMACR and ZFPL1 consistently demonstrated superior discrimination (**Figures 7C–E**). At 1 year postoperatively, the combined model achieved an AUC of 0.96, exceeding that of AMACR alone (AUC = 0.93) and ZFPL1 alone (AUC = 0.91). At 2 years, the AUC increased to 0.97, outperforming AMACR (AUC = 0.95) and matching the performance of ZFPL1 alone (AUC = 0.97). At 3 years, the combined model maintained stable predictive accuracy with an AUC of 0.95, remaining superior to either biomarker used individually.

Overall, the combined model exhibited consistently high AUC values (approximately 0.95) across 1-, 2-, and 3-year follow-up periods, indicating good discriminative ability within this single-center cohort. However, given the relatively small sample size and limited number of recurrence events, these time-dependent AUC estimates may be subject to optimism bias. Accordingly, the results should be interpreted with caution and require confirmation in larger, independent validation cohorts.

### Postoperative survival outcome analysis

**Figure 8:**
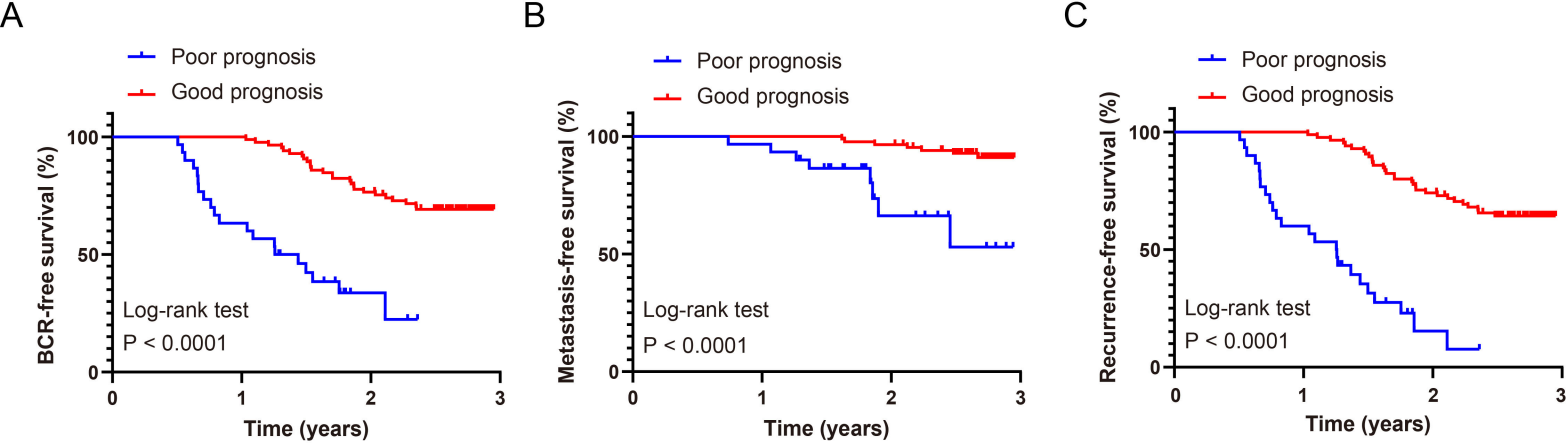
Kaplan–Meier survival curves stratified by prognostic risk group. **(A)** Biochemical recurrence-free survival (BCRFS), with biochemical recurrence as the endpoint. **(B)** Metastasis-free survival (MFS), with the first occurrence of distant metastasis as the endpoint. **(C)** Recurrence-free survival (RFS), with a composite endpoint of biochemical recurrence, distant metastasis, or prostate cancer–specific death.

Biochemical recurrence (BCR), BCR-related metastasis, and PCa-related death were evaluated using biochemical recurrence-free survival (BCRFS), metastasis-free survival (MFS), and RFS, respectively. Kaplan–Meier analyses demonstrated significantly worse survival outcomes in the recurrence group compared with the non-recurrence group. For BCRFS, the log-rank test was significant (χ^2^ = 37.13, *p* < 0.0001), with HR = 4.94 (95% CI: 2.12–11.52). The median BCRFS was 1.35 years in the recurrence group and was not reached in the non-recurrence group (**Figure 8A**). For MFS, the between-group difference was also significant (χ^2^ = 18.33, *p* < 0.0001; HR = 6.57, 95% CI: 1.58–27.27), and the median MFS was not reached in either group (**Figure 8B**). Using a composite endpoint of BCR, distant metastasis, or PCa–related death, RFS was significantly shorter in the recurrence group (χ^2^ = 60.29, *p* < 0.0001; HR = 6.12, 95% CI: 2.66-14.08). The median RFS was 1.26 years in the recurrence group and was not reached in the non-recurrence group (**Figure 8C**).

### AMACR and ZFPL1 are highly expressed in PCa tissues

**Figure 9:**
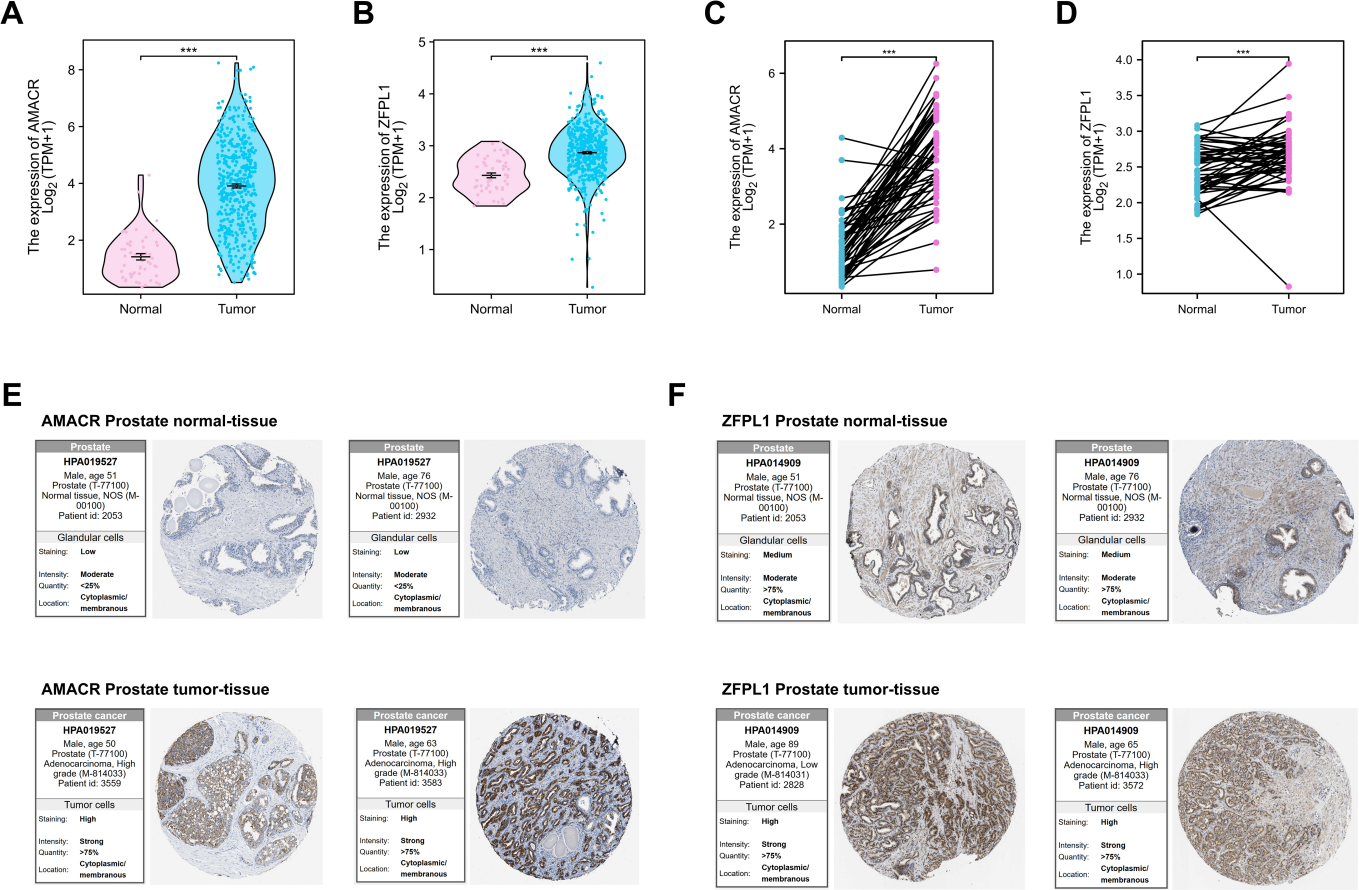
Expression patterns of AMACR and ZFPL1 in prostate cancer and normal tissues. **(A, B)** mRNA expression levels of AMACR **(A)** and ZFPL1 **(B)** in tumor (n = 501) and normal (n = 52) prostate tissues from the TCGA dataset. **(C, D)** Paired analysis of AMACR **(C)** and ZFPL1 **(D)** mRNA expression in matched tumor and normal prostate tissue samples (n = 52 pairs) from the TCGA dataset. **(E, F)** Representative immunohistochemistry images of AMACR **(E)** and ZFPL1 **(F)** protein expression in prostate cancer and normal tissues obtained from the Human Protein Atlas. ***p < 0.001 for between-group comparisons.

To further investigate the expression profiles of AMACR and ZFPL1 in PCa, transcriptomic data from the TCGA-PRAD cohort were analyzed. As shown in **Figures 9A, B**, both AMACR and ZFPL1 exhibited significantly higher mRNA expression levels in tumor tissues compared to normal prostate tissues (*p <* 0.001). Paired analyses further confirmed that AMACR and ZFPL1 expression was consistently elevated in tumor tissues relative to matched adjacent normal tissues from the same patients (**Figures 9C, D**, p *<* 0.001), suggesting a close association with tumorigenesis. These findings were further supported by protein-level validation using immunohistochemistry data from HPA. AMACR exhibited weak or negative cytoplasmic staining in normal prostate tissues but strong positivity in tumor samples. Similarly, ZFPL1 showed low expression in normal tissues and moderate to strong staining in PCa specimens (**Figures 9E, F**).

## Discussion

This study demonstrates a significant association between serum AMACR and ZFPL1 levels and clinical outcomes following RP in patients with PCa. TMultivariable logistic regression identified age, tumor stage, histological differentiation, and Gleason score as independent predictors of recurrence. By integrating these clinicopathological factors with serum AMACR and ZFPL1 levels, we developed a risk nomogram that showed strong discriminative performance for predicting postoperative recurrence. Notably, elevated serum AMACR and ZFPL1 levels were independently associated with an increased risk of adverse outcomes, supporting their potential utility as clinically relevant prognostic biomarkers. Collectively, these findings suggest that the proposed nomogram may serve as a practical tool for individualized risk stratification and postoperative management in PCa.

From a metabolic perspective, AMACR is a key enzyme in branched-chain fatty acid metabolism. Aberrant activation of AMACR can reprogram tumor lipid metabolism, enhancing energy production and biosynthetic capacity to support tumor cell survival and proliferation after surgery (20–22). This metabolic reprogramming enables residual tumor cells to maintain a proliferative advantage after surgery and promote the production of pro-inflammatory mediators, such as prostaglandin E2, thereby reshaping the immune microenvironment and facilitating immune evasion (23–25). In addition, AMACR overexpression may disrupt androgen receptor (AR) signaling homeostasis, increasing the adaptability of castration-resistant tumor clones (26). This mechanism may partly explain AMACR’s unique ability to predict long-term recurrence.

ZFPL1 primarily functions in the regulation of intracellular organelle networks. As a key factor in maintaining Golgi apparatus integrity (27), ZFPL1 modulates the processing and transport of secretory proteins, thereby influencing the release efficiency of pro-metastatic factors such as matrix metalloproteinases (MMPs) and vascular endothelial growth factor (VEGF) (28). In addition, its zinc finger domain may participate in chromatin remodeling complexes, contributing to epigenetic regulation, including H3K27me3-mediated silencing of epithelial markers such as E-cadherin, which accelerates epithelial–mesenchymal transition (EMT) (29). Through its combined roles in secretory regulation and epigenetic remodeling, ZFPL1 may act as a molecular hub linking intrinsic tumor cell properties with dynamic interactions in the tumor microenvironment. Notably, ZFPL1 has been associated with the neuroendocrine phenotype, a highly aggressive subtype of PCa characterized by rapid systemic dissemination (30, 31). Moreover, AMACR expression has also been reported in gastric neuroendocrine tumors (32). Together, these findings support further investigation of the prognostic significance of ZFPL1 and AMACR in aggressive forms of PCa.

The synergistic interaction between AMACR and ZFPL1 may arise from a positive feedback loop linking metabolic reprogramming with secretory regulation. AMACR-driven lipid oxidation supports membrane biogenesis and energy supply required for enhanced secretory activity, whereas ZFPL1-regulated factors, such as IGF-1, activate the PI3K–AKT–mTOR signaling pathway, thereby further amplifying metabolic dysregulation (28). In parallel, AMACR may disrupt AR signaling and promote castration resistance (33), whereas ZFPL1 supports Golgi apparatus integrity, modulates the secretion of pro-invasive factors including VEGF and MMPs, and facilitates EMT through H3K27me3-mediated repression of E-cadherin. This dual-axis regulatory framework integrates intrinsic tumor cell autonomy with microenvironmental adaptation and may explain the enhanced prognostic performance of the combined biomarker model. Consistently, the integrated model demonstrated high accuracy at 1-, 2-, and 3-year postoperative intervals (AUC > 0.95), significantly outperforming conventional models based on TNM stage, Gleason score, or CAPRA nomogram (6), especially in patients with negative imaging but elevated molecular recurrence risk.

While several emerging biomarkers, such as PCA3, KLK2, PEDF (34), lncRNA ProsRISK score (35), and multi-protein panels including FN1 and MMP9, have shown potential for predicting recurrence, many remain in early developmental stages or require complex multicenter validation. In contrast, AMACR, a well-characterized enzyme involved in lipid metabolism, has demonstrated high specificity and sensitivity across prostate tumor tissues and multiple body fluids, including urine, semen, and blood (36, 37). ZFPL1, a recently identified regulator of secretion and epigenetic remodeling, is highly expressed in neuroendocrine PCa, extending the mechanistic landscape beyond lipid metabolism. Importantly, the ELISA-based detection of both biomarkers offers operational simplicity, low cost, and scalability, supporting their feasibility for routine clinical use, including primary care settings and telemedicine-based postoperative follow-up. Furthermore, analyses of data from TCGA and HPA confirmed significantly elevated mRNA and protein expression of AMACR and ZFPL1 in tumor tissues compared with normal prostate tissues, with consistent upregulation observed in paired samples. Together, these findings highlight AMACR and ZFPL1 as a mechanistically complementary, clinically robust, and technically accessible biomarker pair with strong translational potential for postoperative risk stratification in PCa.

The clinical application of this model must account for both population-specific characteristics and the dynamic progression of the disease. For instance, the strong association between aberrant AMACR expression and aggressive phenotypes in African American men highlights the need to establish ethnicity-specific threshold systems. In addition, postoperative fluctuations in serum AMACR and ZFPL1 levels support the use of adaptive monitoring strategies, such as serial assessments at 3, 12, and 24 months after surgery, to capture molecular recurrence trajectories and optimize the timing of clinical interventions. Compared with conventional prognostic tools, the novelty of this model lies in its integration of complementary biomarkers reflecting distinct biological processes: metabolic reprogramming (AMACR) and epigenetic and microenvironmental regulation (ZFPL1). While AMACR reflects tumor dependence on altered energy metabolism, ZFPL1 captures dynamic remodeling of the tumor microenvironment. This multidimensional framework enables a more comprehensive characterization of biological heterogeneity in residual disease, particularly in patients with negative imaging findings but persistent molecular activity. Consequently, the model may facilitate earlier identification of occult micrometastatic disease and provide a critical window for timely intervention. Moreover, the noninvasive nature of serum-based testing allows seamless integration into longitudinal surveillance protocols, supporting individualized follow-up based on biomarker trajectories while potentially reducing healthcare costs and improving monitoring efficiency. From a translational perspective, emerging small-molecule inhibitors targeting AMACR and Golgi-directed strategies involving ZFPL1 represent promising approaches to disrupt the metabolism–secretion axis underlying metastatic progression. Together, these advances suggest a shift in postoperative PCa management from morphology-based staging toward mechanism-driven precision strategies.

This study has several important limitations. First, as a single-center retrospective cohort with a relatively small sample size (n = 115) and no *a priori* sample size or power calculation, the analysis is susceptible to selection bias and model overfitting. In addition, the study protocol was not prospectively registered, and the findings require confirmation in prospectively registered cohorts. Second, model validation was limited to internal cohort splitting (stratified 7:3 into training and test sets), without independent external or prospective validation, which limits the generalizability of the nomogram across institutions and populations. Third, follow-up was largely restricted to the first 3 years after surgery and may not fully capture long-term recurrence patterns or survival outcomes. Future studies should assess the prognostic utility of serum AMACR and ZFPL1, as well as the robustness and calibration of the proposed model, in larger multicenter prospective cohorts encompassing diverse ethnic populations and clinically relevant subgroups, with recalibration performed as necessary.

Notably, the nomogram and the AMACR/ZFPL1-based time-dependent ROC analyses yielded AUC values approaching or exceeding 0.90 at multiple time points. Although this may reflect clear separation between risk groups and relatively uniform management within a single center, such high performance in a dataset with only 30 adverse events is likely influenced by optimism and potential overfitting. Despite the use of bootstrap-based internal validation, both the nomogram and the AMACR/ZFPL1 model should be regarded as exploratory until they are externally validated and, if necessary, recalibrated.

## Data Availability

The raw data supporting the conclusions of this article will be made available by the authors, without undue reservation.

## Supplementary material

Supplementary Figure 1 The overall workflow.
