# Supplementary figures and images for: AMACR and ZFPL1 serum biomarkers enhance precision in predicting postoperative prostate cancer outcomes
Source: Front Oncol. 2026 Feb 17;16:1625125. doi: 10.3389/fonc.2026.1625125 (PMC12953113; doi:10.3389/fonc.2026.1625125)

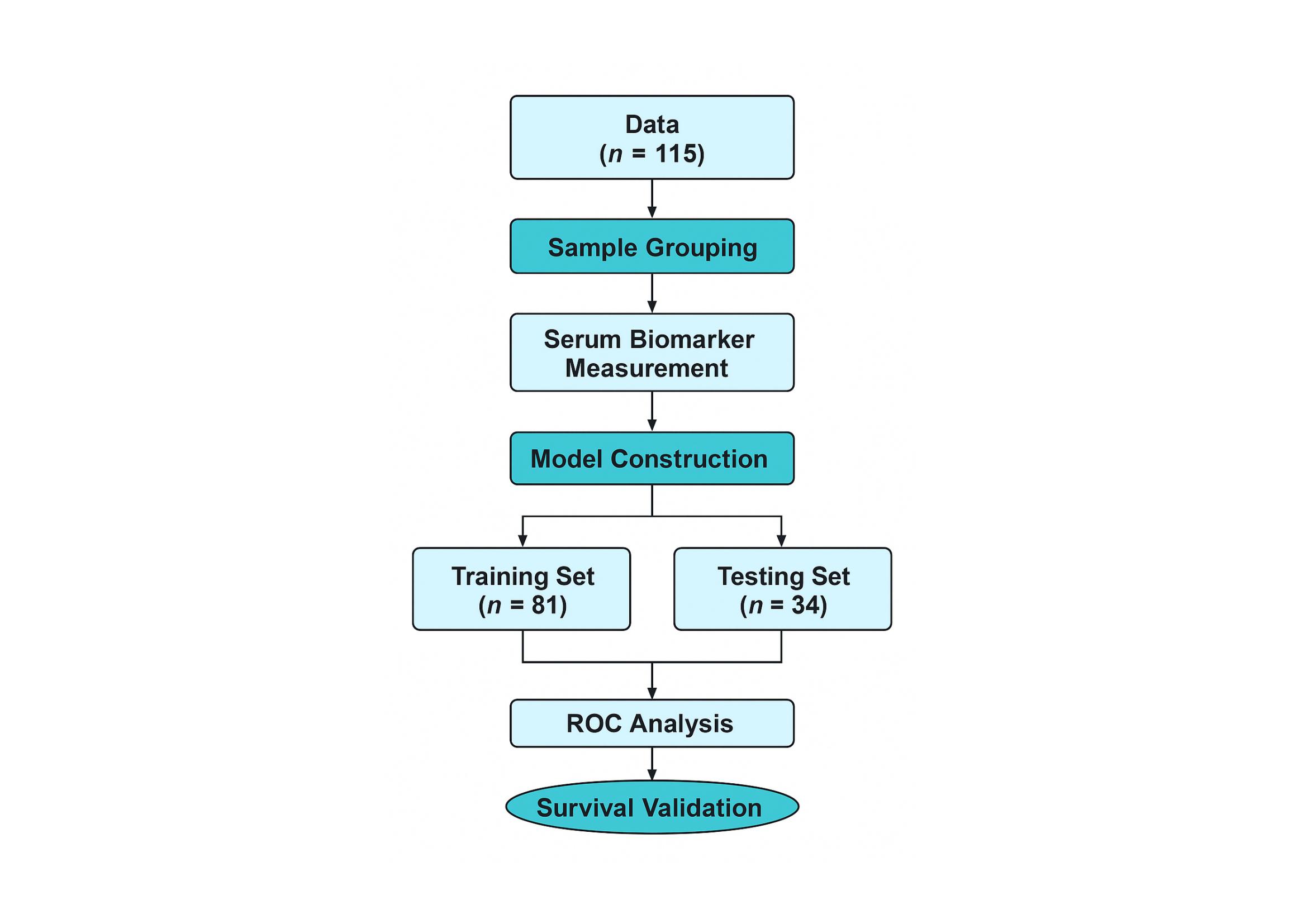

Supplement: Supplementary Figure 1 — The overall workflow. [file Image1.jpeg]
